# Exploring the Full Potential of Radiofrequency Technology: A Practical Guide to Advanced Radiofrequency Ablation for Complex Ventricular Arrhythmias

**DOI:** 10.1007/s11886-024-02048-z

**Published:** 2024-05-03

**Authors:** J. B. Tonko, P. Lambiase

**Affiliations:** 1 Institute of Cardiovascular Science, University College London, 62 Huntley St, London, WC1E 6DD UK; 2 Bartholomew s Hospital, W Smithfield, London, UK

**Keywords:** Ventricular tachycardia, Radiofrequency ablation, Epicardial ablation, Bipolar ablation, Impedance modulation, Irrigated RF needle, Intramyocardial RF delivery, Multipolar RF ablation

## Abstract

**Purpose of Review:**

Percutaneous radiofrequency (RF) catheter ablation is an established strategy to prevent ventricular tachycardia (VT) recurrence and ICD shocks. Yet delivery of durable lesion sets by means of traditional unipolar radiofrequency ablation remains challenging, and left ventricular transmurality is rarely achieved. Failure to ablate and eliminate functionally relevant areas is particularly common in deep intramyocardial substrates, e.g. septal VT and cardiomyopathies. Here, we aim to give a practical-orientated overview of advanced and emerging RF ablation technologies to target these complex VT substrates. We summarize recent evidence in support of these technologies and share experiences from a tertiary VT centre to highlight important “hands-on” considerations for operators new to advanced RF ablation strategies.

**Recent Findings:**

A number of innovative and modified radiofrequency ablation approaches have been proposed to increase energy delivery to the myocardium and maximize RF lesion dimensions and depth. These include measures of impedance modulation, combinations of simultaneous unipolar ablations or true bipolar ablation, intramyocardial RF delivery via wires or extendable RF needles and investigational linear or spherical catheter designs. Recent new clinical evidence for the efficacy and safety of these investigational technologies and strategies merits a re-evaluation of their role and clinic application for percutaneous VT ablations.

**Summary:**

Complexity of substrates targeted with percutaneous VT ablation is increasing and requires detailed preprocedural imaging to characterize the substrate to inform the procedural approach and selection of ablation technology. Depending on local experience, options for additional and/or complementary interventional treatments should be considered upfront in challenging substrates to improve the success rates of index procedures. Advanced RF technologies available for clinical VT ablations include impedance modulation via hypotonic irrigation or additional dispersive patches and simultaneous unipolar as well as true bipolar ablation. Promising investigational RF technologies involve an extendable needle RF catheter, intramyocardial RF delivery over intentionally perforated wires as well as a variety of innovative ablation catheter designs including multipolar linear, spherical and partially insulated ablation catheters.

## Introduction

**Fig. 1:**
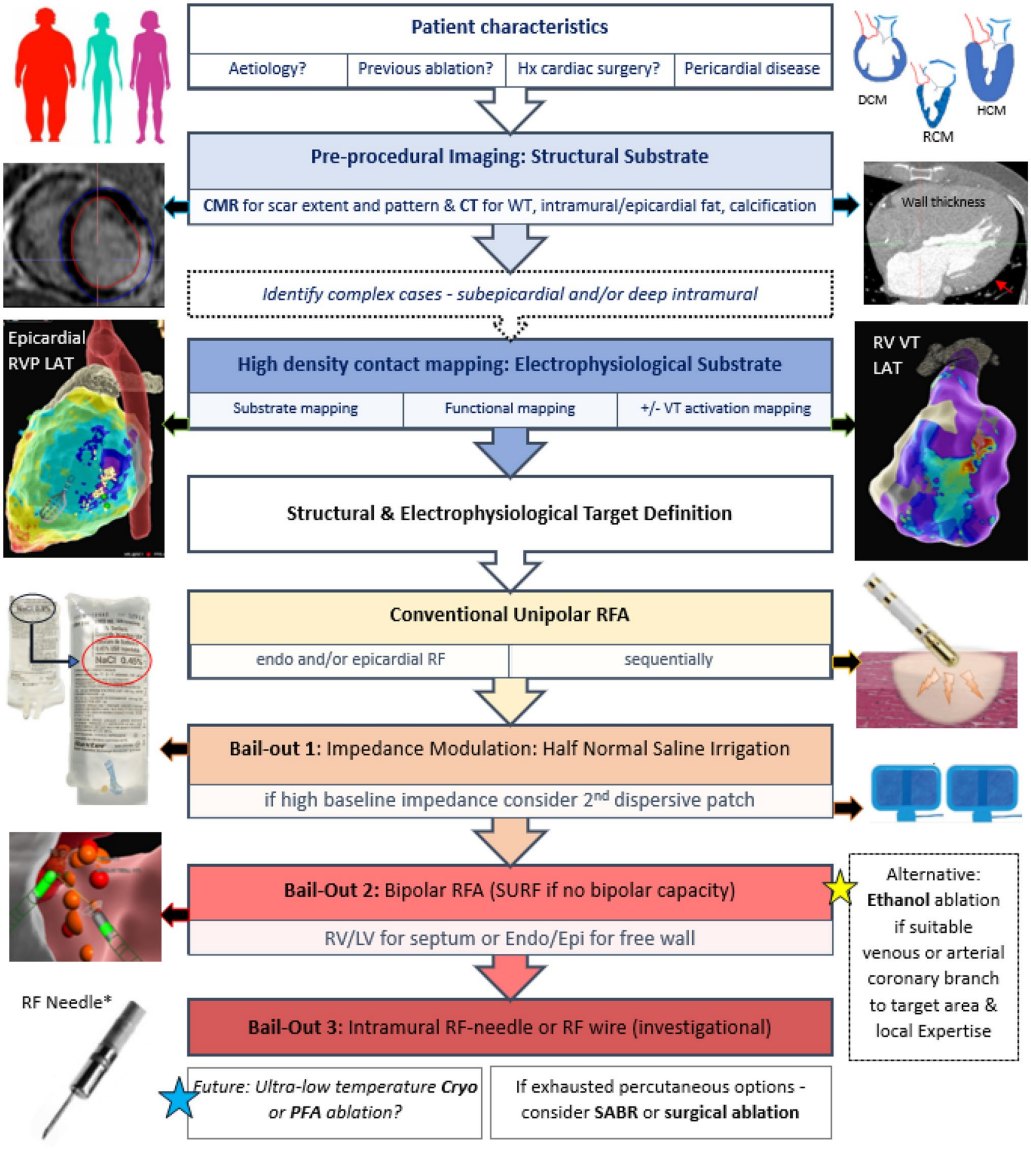
Workflow for advanced ventricular tachycardia ablation. (*Irrigated RF needle reprinted from Stevenson W et al. J Am Coll Cardiol 2019; 73(12): 1413–1425, with permission from Elsevier) [25]

Ventricular tachycardias and ICD shocks for ventricular arrhythmias are predictive of subsequent heart failure hospitalization, impaired quality of life and death [1–3]. Percutaneous catheter ablation for VT is a commonly used adjunctive treatment modality complementing anti-arrhythmic medication and device therapy to prevent recurrent VT and ICD shocks. Several recent randomized trials reconfirmed the successful reduction of VT burden and ICD therapies by ablation [4, 5]. Yet, prognostic benefit remains an area of debate with only one secondary prevention trial showing mortality benefit after extensive ablation (PARTITA) [6]. The lack of definite prognostic benefit and comparatively high arrhythmia recurrence rates, e.g. in cardiomyopathies, highlights the need for improved ablation technology to target all functionally critical areas, particularly if deep intramural, “difficult-to-reach” and/or very extensive arrhythmogenic substrates are present. Figure 1 provides a summary workflow of how to prepare and manage these challenging cases using advanced RF ablation techniques. Alternative and/or complementing novel non-RF technologies for VT ablation, including cryo- and pulse field ablation, chemical ethanol ablation, neuromodulation and stereotactic ablative radiotherapy, have recently been reviewed [7].

## Characterization of VT Substrate for Procedural Planning

**Fig. 2:**
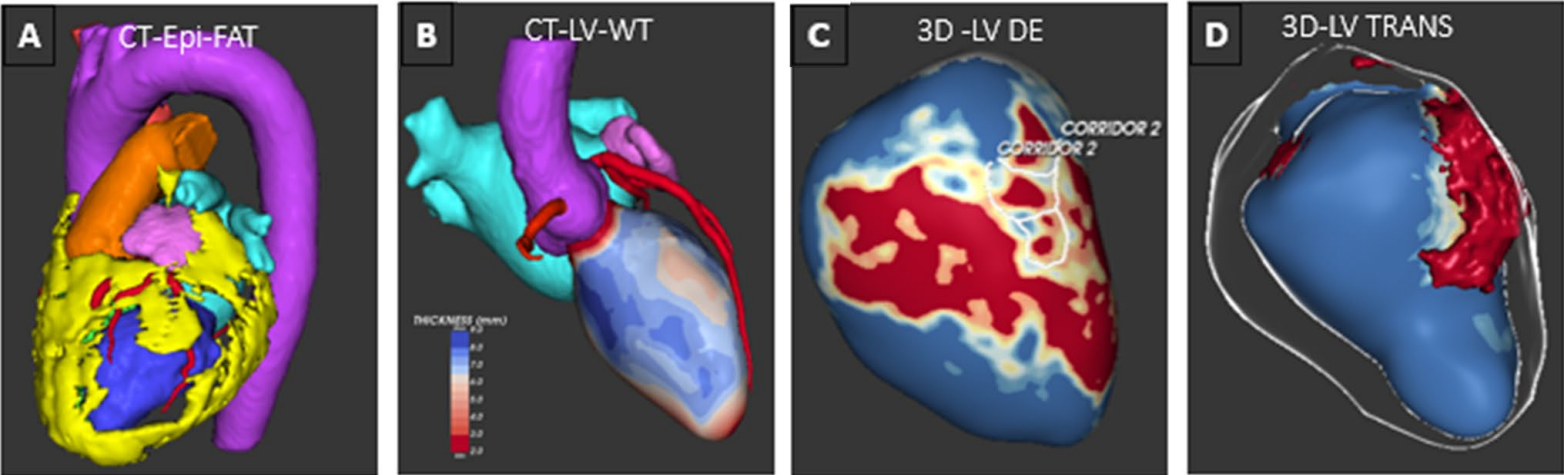
Structural substrate characterization: clinical imaging modalities to guide procedural planning (here, visualized in Adas3D; V2.11.0 2022, Galgo Medical, Spain). **A** Anatomical cardiac CT segmentation and extensive epicardial fat (yellow). **B** CT left ventricular wall thickness model combined with anatomical segmentation of aorta, coronaries and left atrium. **C** 3D CMR LGE scar model depicting scar core in red, border zone in white and healthy tissue in blue. Possible conductive channels are localized per myocardial layer and outlined by white lines. **D** Visualization of transmurality of scar in 3D CMR LGE model

Given the heterogeneity of underlying structural and electrophysiological alterations implicated in ventricular arrhythmogenesis, the characterization of the individual substrate by means of non-invasive imaging (Fig. 2) is important to determine a suitable ablation approach. Cardiac MRI is considered the gold standard for non-invasive identification of myocardial scar tissue and is vital in identifying patients with a difficult-to-reach substrate in whom conventional RFCA has a high failure rate [8]. The high spatial resolution of cardiac CT scans makes it an equally valuable alternative indicating myocardial scarring by identifying areas of abnormal wall thinning of < 5 mm [9] and delayed iodine enhancement [10]. Areas of relatively preserved wall thickness separating areas of thinning within the myocardial scar have been found to correlate to critical target sites for VT ablation [11, 12]. Visualization and quantification of intramural and epicardial fat are also useful. It has been suggested that critical VT corridors may traverse infarcted tissue through or near areas of lipomatous metaplasia (LM) due to higher regional resistance and reduced current loss as the impulse travels [13]. In addition, LM has been associated with increased repolarization dispersion within post infarct VT circuit sites [14]. Lastly, insulating layers of intramural fat are a known limitation for transmural lesion formation with RF ablation, whereas epicardial fat > 3 mm renders RF ablation less effective during epicardial ablations [15]. Epicardial fat may also impact optimal voltage thresholds to identify abnormal substrates [16].

**Fig. 3:**
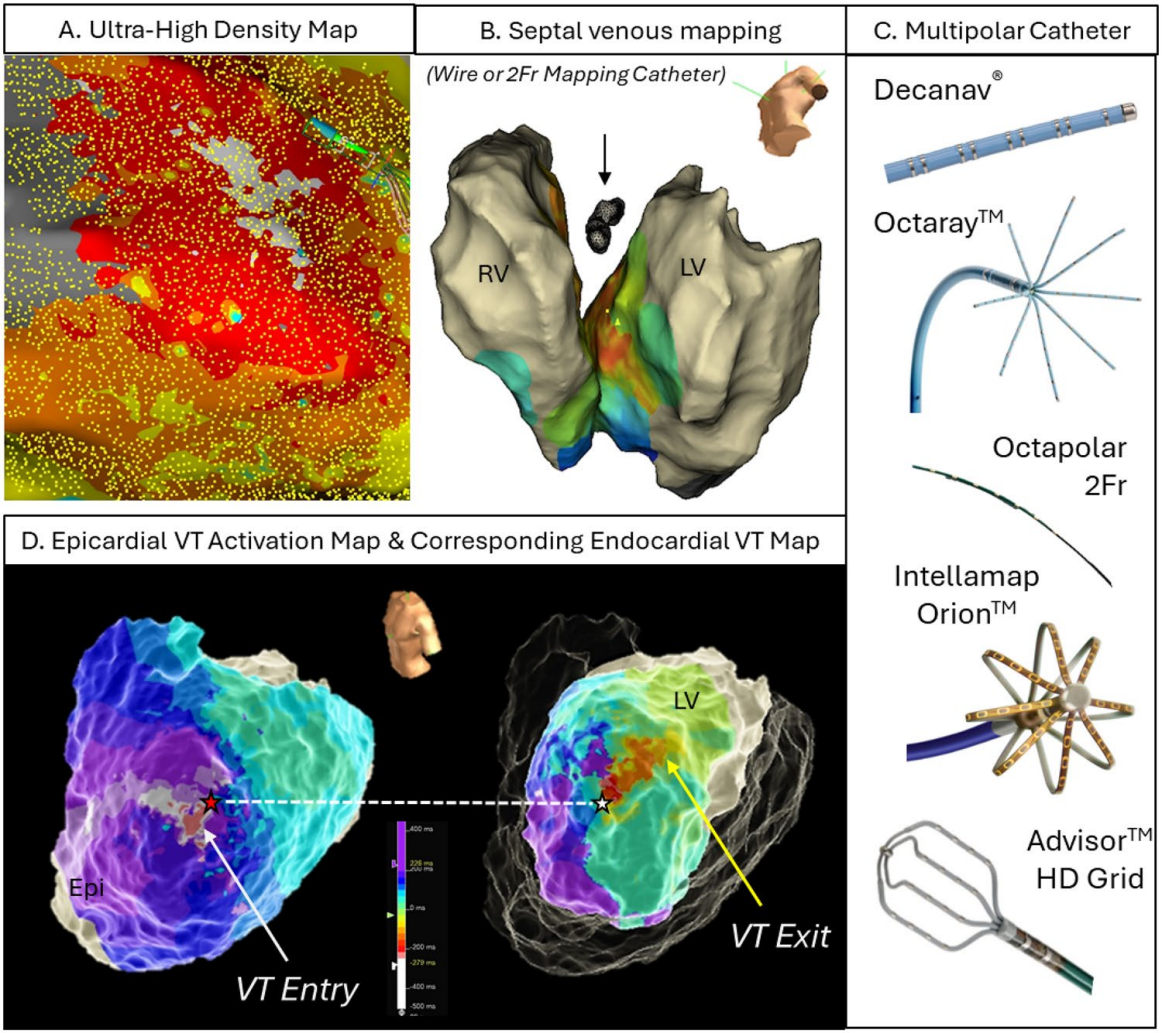
Electrophysiological substrate characterisation and activation mapping: **A** ultra-high-density substrate and activation mapping using 3D electro-anatomical mapping systems (yellow dots representing collected EGMs—collection of several thousand mapping points has become the norm in many VT ablation procedures). **B** Intramural mapping over coronary venous system. **C** Multipolar mapping catheter in a variety of configurations and shapes with small electrodes allowing for high resolution and rapid data collection to guide VT ablations. **D** Combined endo-epicardial mapping to facilitate identification of 3-dimensional activation patterns—here, example of ischemic cardiomyopathy patient with large transmural anterior scar with VT entry site at epicardial anterior LV (white/red = entry) diving intramurally (indicated by red star) and re-emerging on opposing endocardial surface (indicated by white star) where it exits

Improved preprocedural imaging is complemented by the substantial progress in contact mapping technology using multipolar mapping catheters with small closely spaced electrodes (Fig. 3). Also, a variety of functional mapping protocols to identify zones of deceleration [17], fractionation [18], rotational activation [19] and decremental conduction [20] to delineate possible target zones have been proposed. In selected patients, a combined endo-epicardial approach may be preferred as a first-line strategy in non-ischemic/arrhythmogenic but also ischemic cardiomyopathies [21, 22]. Randomized controlled trials are ongoing to assess the benefit of such a more “aggressive” approach (clinicaltrials.gov, NCT02358746) [23]. Strategies for intramural mapping over the coronary venous or arterial system with small-sized linear mapping catheters, wires, or specially designed needles have been proposed to assess deep septal substrate [24].

## Advanced Radiofrequency Techniques: Exploring the Full Potential of RF Ablation

### Impedance Modulation

#### Rationale

**Fig. 4:**
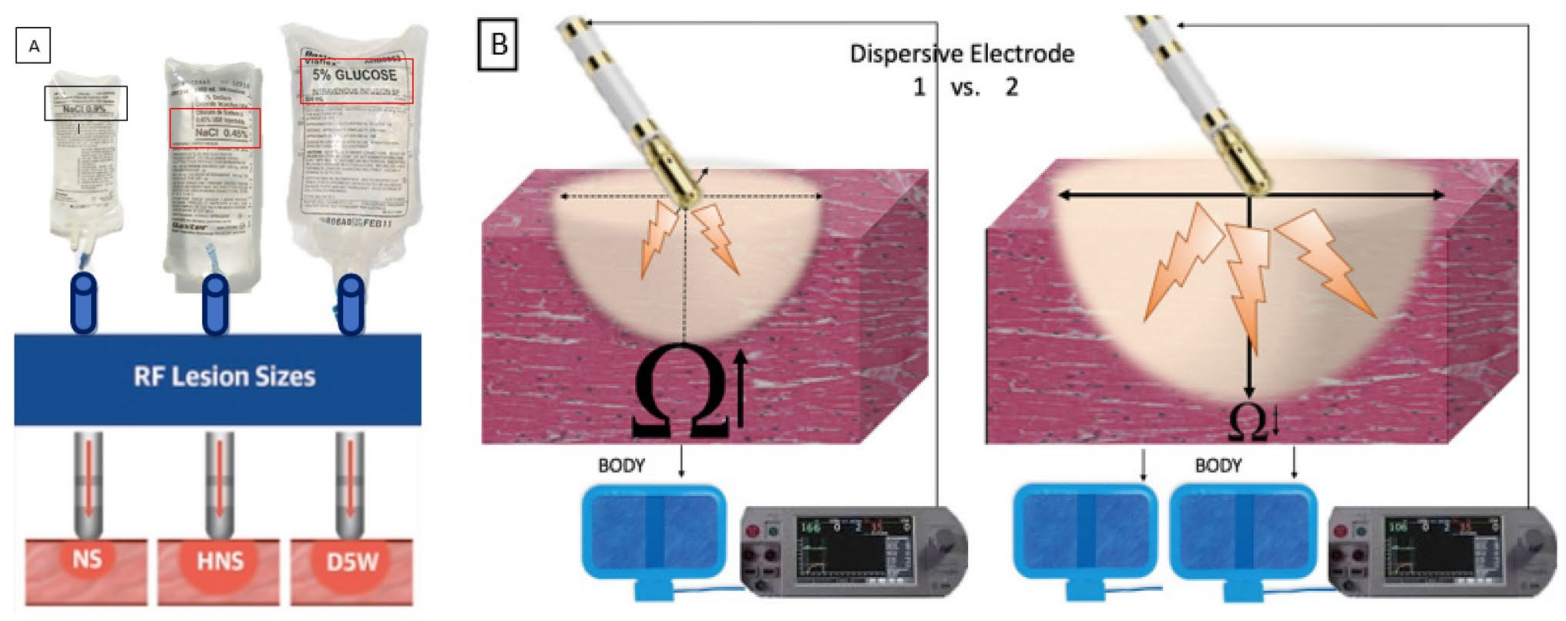
Impedance modulation options: **A** hypotonic (middle bag with red box highlighting NaCl 0.45%) and non-ionic irrigation (bag on right with red box highlighting 5% Glucose) of ablation catheters increase impedance surrounding the catheter tip to facilitate directing current into the myocardium and increase lesions size. (adapted from Bennett R et al. JACC Clin Electrophysiol. 2021 Oct;7(10):1229–1239, with permission from Elsevier) [38]. **B** Additional dispersive patches placed on the back, thigh or leg of the patient can lower the system impedance to improve energy delivery to the tissue and facilitate lesion formation

Traditional unipolar RF ablation applies alternating current from the tip of the ablation catheter, from where it is transmitted through myocardial tissue, blood and the body to the dispersive electrode on the skin and returned to the RF generator. To prevent excessive heating at the tip-tissue interface and the risk of char formation limiting the delivery of RF energy, ablations in the ventricle are commonly performed using irrigated ablation catheters employed with power-controlled modes. This has been associated with deeper and larger lesions compared to non-irrigated catheters [26]. Yet the power set on the RF generator is not equivalent to the actual energy delivered to the tissue which defines the extent of tissue heating and necrosis (and therefore scar formation). The latter is substantially affected by the baseline impedance with higher baseline impedances associated with less effective lesions. Based on this biophysical rationale, impedance modulation strategies to increase lesion dimensions and, ideally, durability have been proposed [27, 28]. A major advantage of impedance modulating approaches is their ease of implementation in the standard EP lab without the requirement of special equipment (see illustration in Fig. 4).

#### How Does One Modify Impedance in the EP Lab?

##### Hypotonic Irrigation

Traditionally open-irrigated catheters use 0.9% saline solutions. Hypotonic 0.45% (half normal) saline has a relatively higher impedance (around 180 Ohm vs. 90 Ohm of normal saline) and, if replaced as the catheter irrigant, can increase the impedance of the irrigation fluid surrounding the ablation tip. The latter minimizes the dispersion of current into the blood pool and enhances the RF current delivery into the myocardium. This has been reported to result in deeper ablation lesion dimensions for the same delivered power in high-flow open-irrigated catheters ex vivo and in vivo [29]. Ex vivo studies using infra-red thermal imaging for lesion characterization confirmed this finding [30]. In contrast, animal studies using low-flow (15 ml/min) open-irrigated ablation catheters in beating hearts found that the use of HNS irrigant resulted in greater heating but not significantly increased lesion size [31]. This conflicting result of the latter study has been hypothesized to be due to a premature termination in the HNS group because of higher rates of steam pops in the context of low-flow irrigation as well as a lower force-time integral [32]. Clinical data from a prospective multi-centre trial showed HNS to be effective for PVC/VT ablation sources (use with power settings titrated up to 50 W for > 60 s), including cases with previously failed standard RF ablation. A comparatively high steam pop rate of 12.6% was reported but no cardiac perforation [33]. In turn, a more recent multi-centre trial for half normal saline vs. normal saline irrigation in outflow tract arrhythmias showed similar success and safety (steam pop 2.4% in HNS vs. 1.2% in NS) but shorter total ablation time with HNS [34]. For epicardial ablation, it has been suggested that HNS may (theoretically) be protective of parietal pericardium injury from heating if appropriate catheter orientation towards the epicardium can be achieved.

##### Non-ionic Irrigation

A more aggressive approach consists of employing entirely non-ionic irrigation with, e.g. dextrose-5 [35], causing significantly larger lesions than both HNS and NS at the cost of a high rate of steam pops (if applied in a perpendicular catheter position, but not in parallel position) [27]. Clinical use of non-ionic irrigation is limited to case reports employing it successfully in combination with bipolar RF [36].

##### Dispersive Patch Location and Number

Return patches are traditionally placed on the left thigh or left flank. Addition of a second dispersive patch and/or moving them closer to the chest to reduce baseline impedance has been proposed for ventricular ablation after failed response to standard RFA and/or use of low-ionic irrigants. A single-centre experience reported a baseline impedance reduction of 23 Ohm associated with a significant increase in current output with the use of additional return electrodes (thigh and flank, flank and scapula, flank and scapula and sternum; power 30–50 W, CF > 5 g, application duration 1–2 min). Steam pop rate was not significantly different between higher and lower impedance settings (7.1 vs. 8.2%). Yet the steam pop rate significantly increased in the subgroup with additive return electrode in combination with the use of HNS as well as in lesions with impedance drop of > 14 Ohm within the first 10 s [37].

#### Practical Considerations

The biophysical rational has been supported by clinical evidence that there is an additive therapeutic value in using impedance modulating measures to facilitate larger lesion formation. This is of particular interest in areas of thick myocardium with deep substrate, e.g. mid-septal, LV summit or a subset of papillary muscle arrhythmias. Yet, in clinical practice, multiple parameters influence final lesion size: catheter tip size, tissue orientation, contact force, stability, irrigation flow rate, lesion duration, power settings and underlying tissue characteristics (fibrosis, fat, calcifications etc.) all impact lesion dimensions in addition to above-discussed baseline impedance. Also, the differences between endo- and epicardial biophysics of ablation have been highlighted [39]. Lastly, it has to be noted that traditional ablation lesion indices (“AI”, “LSI”) are not validated to guide ablation with hypotonic irrigation although this might be less relevant in the ventricle where it has recently been shown to have limited value in any case [40].

Increased energy delivery to the tissue comes with a concern of an increase in adverse events supported by the higher rate of steam pops in the literature. Continuous monitoring of impedance changes is mandatory. Particularly, a rapid drop in the first 10 s needs to alert the operator to a high steam pop risk. A combination of impedance modulating methods (additive dispersion patches and hypotonic irrigations) appears to be associated with an unacceptable high risk of steam pops. More precise methods for estimating lesion formation in real time are required for the safe delivery of impedance modulation [41].

Overall, the absolute additive benefit for patient outcome remains open, and more evidence characterizing particularly the safety profile of HNS and glucose irrigation in interventional electrophysiology is needed prior to recommending it as a routine strategy for VT ablation. Yet it does have a role as an easily implemented, ubiquitously available bail-out strategy if the ablation of the deep substrate with traditional RFA fails.

### Two-Catheter Approaches: Simultaneous Unipolar (SURF) and True Bipolar RF Ablation (BPA)

#### Rationale

Despite extensive RF ablation with open-irrigated catheters and impedance modulation measures, deep intramural substrate may not be eliminated. Simultaneous unipolar RF ablation over 2 separate ablation systems and bipolar ablation with RF current being applied between 2 ablation catheter tips as part of the same circuit have been proposed to increase current density in the intramural region. A greater rise in the tissue temperature by increased conductive heating in the intramural region due to resistive heating from both sides of the wall may be achieved. Sufficient tissue heating is fundamental to cause irreversible cellular damage and necrosis.

#### How to Use Two Ablation Catheters to Target Deep Substrate?

##### Simultaneous Unipolar RF Ablation (SURF)

Two (open-irrigated) catheters are being placed at anatomically opposite sites over the area of interest and connected to two separate RF generators. Animal and ex vivo studies confirmed that SURF ablation can increase lesion sites [42], and case reports suggested its clinical usefulness for septal ventricular tachycardias [43] and intramural LVOT [44] after prolonged sequential RF failed. In a small case series for the non-ischemic substrate proposed, energy delivery was provided by up to 40 W for up to 3 min and independently titrated for each catheter to achieve an impedance drop of at least 15% of the baseline values. No procedural complications or steam pops were observed with 67% of patients being VT-free after a median follow-up of 20 months [45]. Advantages of the SURF method include detailed monitoring of ablation parameters (impedance, power delivery, stability, irrigation flow) over the separated generators as well as individual independent adjustment of both ablation catheters. Yet only one catheter will be able to be displayed with contact force within the mapping system.

The approach can be employed using generally available equipment in a standard EP catheter lab.

##### True Bipolar RF Ablation (BPA)

In vitro experiments suggested that bipolar ablations may achieve transmurality in segments as thick as 25 mm with impedance drops of 30–40 Ohm with the delivery of up to 2 min and 50 W of BPA. This was compared to unipolar RF, which failed to achieve transmurality in segments thicker than 15 mm [46]. Other studies aiming to characterize bipolar lesion formation showed deeper but not wider lesions with BPA [47]. Catheter tip orientation (parallel or perpendicular) and type (electrode size, irrigation) were found to be important determinants for bipolar lesion size. Largest and deepest lesions were achieved using 2 irrigated catheters orientated perpendicular to the surface without causing a statistically higher risk of steam pops [48]. Yet a separate study specifically evaluating the size of the return electrode tip (3.5 vs. 8 mm) achieved significantly larger and more commonly transmural lesions if an 8-mm tip was placed in *parallel*, and longer applications (up to 240–300 s) were being delivered [49]. In turn, small tip catheters (3–5 mm) were associated with higher impedance values and have been hypothesized to achieve a more dense lesion core and possibly less collateral damage, thus allowing more selective targeting of an area of interest [50].

The first clinical case series of septal and free wall VT using percutaneous irrigated BPA described the left-sided catheter as the active open-irrigated ablation catheter connected to the RF generators. The right-sided catheter was connected to the indifferent electrode connection by using a custom cable in that the distal pole of the right-sided catheter acts as the indifferent electrode for the RF current (termed “intracardiac return electrode”, IRE). This setup also allowed us to record the electrograms of the 4 poles [46]. Further case series demonstrated the safety and efficacy in the outflow tracts [51], deep septal circuits [47, 52] and papillary muscle PVCs [53] and most recently for refractory VT requiring endo-epicardial bipolar RFA [54]. Due to the presumed more selective lesion formation, BPA has also been proposed and successfully employed for para-hisian VEs with up to 60 W [55]. Acute success rates were generally high, yet long-term outcome data is still sparse. In one recent study, recurrence rates following bipolar ablation at 12 months have been reported of up to 44% in a population of predominantly non-ischemic deep septal VTs, even though the overall burden was reduced [56]. Case series in patients with therapy refractory ventricular arrhythmias reported freedom from arrhythmia in 52% in a follow-up of up to 2 years after BPA [57•]. The lower effect in bipolar RFA in humans compared to preclinical ex vivo studies has been partially explained by the higher circuit impedance in humans which results in less heating and smaller lesions. For the conductive heating of bipolar ablations to reach the deep intramural sites, longer ablation lesions (up to 2 to 6 min) may be required to reach the deep structures [58].

More recently, a variation of bipolar ablation for deep septal substrate has been proposed involving the placement of small 2Fr octapolar catheters in coronary venous septal perforators and incorporating all intramural mapping electrodes as return electrodes in the ablation circuit. This setup allows delivery of "multipolar" ablation and thus likely achieves higher current density in the area of interest without the requirement of additional equipment other than the 2Fr octapolar catheter. Feasibility and safety were demonstrated in a clinical case report [59].

#### Practical Considerations

**Fig. 5:**
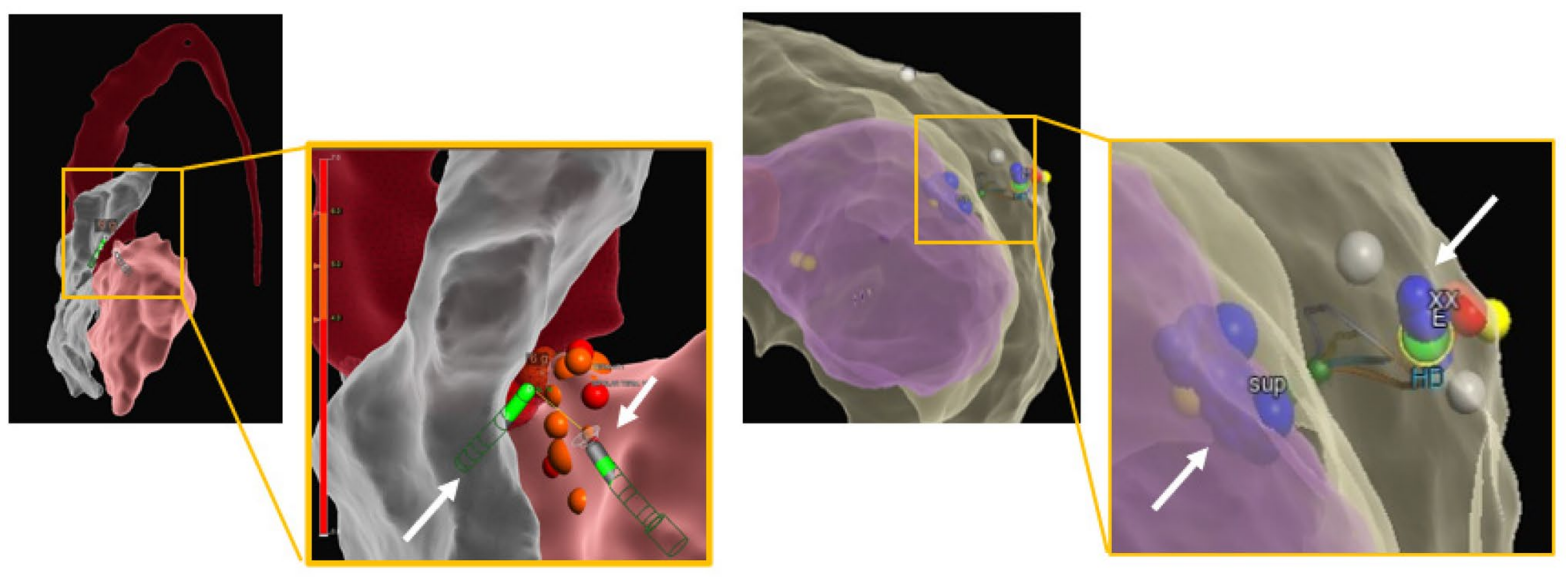
3D electro-anatomical ventricular maps. Left: true bipolar ablation at the septum with active catheter in the LV and return catheter in the RV. Both catheters can be visualized (white arrows pointing at ablation catheter on RV and LV side, respectively), but ablation parameters and contact force will only be displayed for the active catheter. Right: sequential unipolar ablation on the LV lateral wall to target a deep intramural focus on opposing sites of the suspected critical area (white arrows pointing at ablation lesions delivered from endo- and epicardium)

**Fig. 6:**
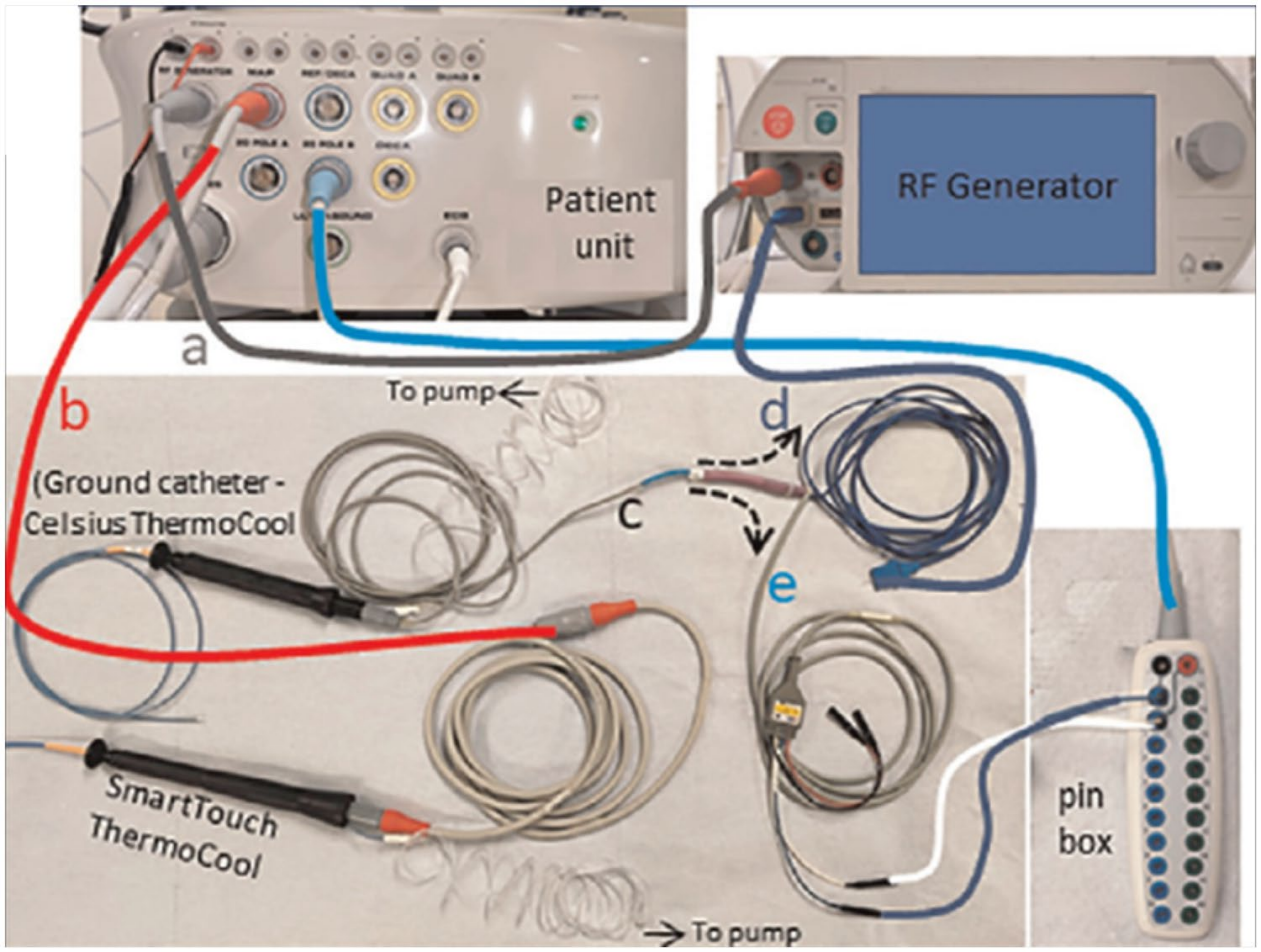
Bipolar setup. Traditionally, the setup for bipolar RF ablation involves a connection of a second ablation catheter over a custom-made cable to a conventional RF generator replacing the standard dispersive patch as the return electrode. (Adapted from Derejko P et al. JACC EP June; 9(6): 733–737, with permission from Elsevier) [54]. New dedicated CE-marked bipolar RF ablation generators have now become available to allow for regular connection of two ablation catheters with separate irrigation pumps as shown by Kany et al. Europace 2022 [57•]

For both SURF and BPA generally, a two-operator approach is required to allow for positioning and maintenance of the stability of the two catheters opposing each other—depending on the target site, this may involve endo- to epicardium, left to right ventricular septum (see Fig. 5), endocardium to coronary vein or pulmonic cusps to coronary cusps. In the case of 3D mapping systems, only one catheter will be able to measure contact force and display a contact vector, whereas the second catheter will be visualized as a diagnostic catheter. For bipolar ablation, impedance, power, stability and temperature monitoring (and adjustment) are only available for one catheter. The custom-made setup and connections and limited monitoring options of ablation metrics for the second ablation catheter have long been a point of criticism. More recently, a standardized setup with a dedicated CE-marked bipolar RFA generator (HAT500, Osypka AG, Rheinfelden, Germany, Fig. 6) has been reported, and feasibility and safety were demonstrated in a multi-centre observational study [57•].

The safety profile of BPA is debated due to conflicting results, as are optimal catheter choice and ablation parameter settings. Some available data suggested that the rate of steam pops may indeed be lower, particularly if careful power titration under strict impedance monitoring and ideally ICE is undertaken [60]. Yet damage to the conduction system with septal ablation and acute occlusion of a coronary artery has been reported [56]. The non-randomized bipolar VT study (NCT02374476) was prematurely terminated after recruiting 49 patients to the bipolar arm due to a high number of serious adverse events including pericardial effusion and cardiac tamponade, complete heart block and pulmonary embolism and recurrent VT in 18% [61]. In turn, in the most recent “real-world” case series employing endo-epicardial bipolar RFA, no serious adverse events were reported [54].

## Ablation Settings: High-Power Long-Duration RF Ablation?

### Rationale

To achieve durable lesion formation, adjusted to the local anatomical characteristics, high-power *short* duration has attracted significant attention for ablation in the mostly thin-walled left atrium to achieve shallow lesions [62]. Yet the role and possible clinical benefit of high-power *long*-duration RF delivery in the thicker ventricular wall is less well characterized. In standard RF settings, maximum lesion size is thought to have reached between 20 and 60 s, yet with irrigated RF systems, lesion growths may still progress beyond 60 s [26]. Also, extending the duration of RF delivery has been shown to partially compensate for difficulties with intermittent tissue contact [63]. High-power (50 W) long-duration (up to 4 min) ablation lesions have been suggested for endocardial ventricular ablation to reach subepicardial substrates [64]. This has been supported by case reports demonstrating the feasibility of epicardial ablation from non-epicardial respectively endocardial sites [65, 66].

#### Practical Considerations

Implementation in the EP lab may be straightforward, but experience with high-power long-duration ablation in the ventricle remains sparse, and further studies are required to characterize lesion formation and clinical utility and safety. If applied, close monitoring of abrupt impedance increase or decrease as well as limiting maximum catheter temperature is advisable in any case. Ablation Lesion Index–guided ablation has been found to be of limited value in scar-related ventricular arrhythmia ablation, did not predict lesion dimensions accurately [40] and can therefore not be recommended to be used as a surrogate to guide ablation duration and power.

## Investigational RF Devices: RF Needles, Wires, Linear, Spherical and Partially Insulated Ablation Catheters

### Rationale

**Fig. 7:**
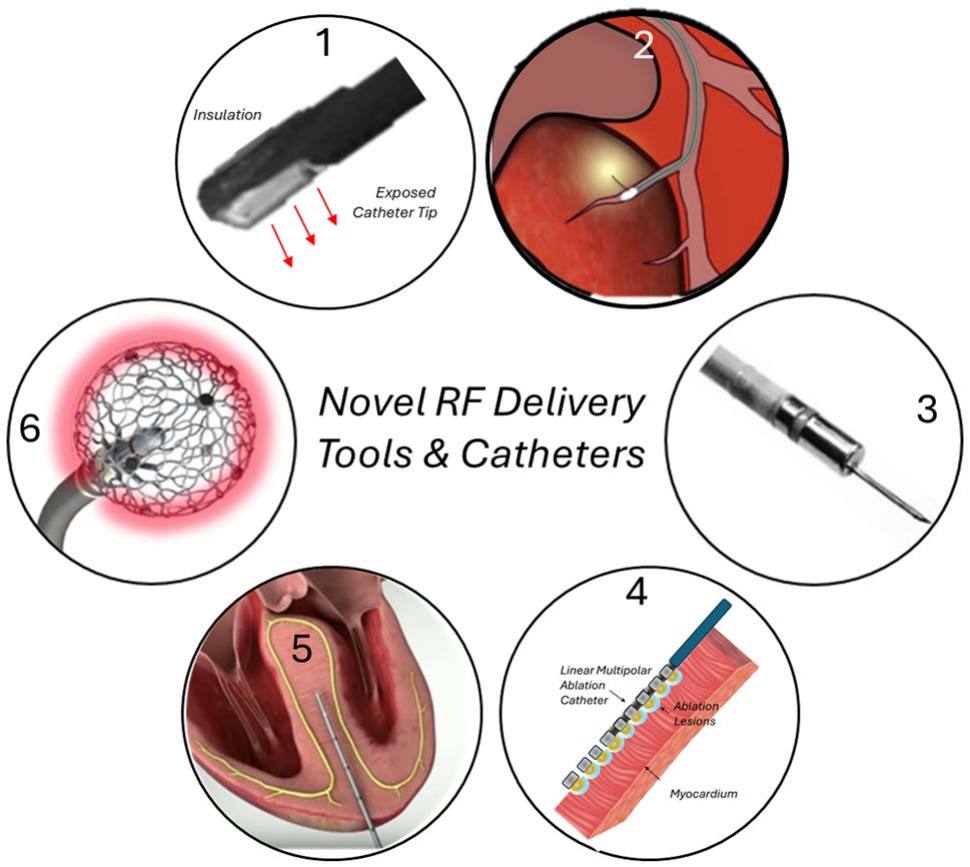
Overview novel and investigational RF delivery tools and catheters. (1) Partially insulated RF ablation catheter tip. (Adapted from Nguyen et al. Heart Rhythm Mar 2015;12(3): 623–630, with permission from Elsevier) [67]. (2) Intentional wire perforation for intramyocardial RF delivery. (Modified and adapted from Romero J et al. Heart Rhythm Case Reports 2018; 4(7): 285–292 with permission from Elsevier)68. (3) Irrigated RF needle catheter. (Adapted from Stevenson W et al. J Am Coll Cardiol 2019; 73(12):1413–1425, with permission from Elsevier) [25]. (4) Schematic illustration of a linear multielectrode ablation catheter (as originally proposed by Nazer B et al.) [69]. (5) Percutaneous intramyocardial septal radiofrequency ablation (PIMSRA)—proposed for RF ablation of hypertrophic obstructive cardiomyopathy. (Adapted from Liu L et al. JACC 2018; 72 (16): 1898–1909, with permission from Elsevier) [70]. (6) Spherical RF catheter (Sphere-9TM Affera Inc., reprinted with permission of Medtronic—currently approved for atrial ablation only). For details, see text

Mapping of ventricular arrhythmias originating deep within the myocardium as well as energy delivery to these areas requires new and innovative approaches. Point-by-point RF ablation is impractical and time-consuming if a substrate homogenization strategy is pursued. New investigational RF devices (illustrated in Fig. 7) attempt to overcome this limitation to rapidly deliver energy over wide areas and deeper if required.

### Investigational Devices

#### Infusion RF Needle

This is a unique method to achieve deeper lesions. The concept was proposed in 2000 [71] followed by animal studies applying different designs and assessing lesion dimensions with variable power and irrigation flow [72–74]. The original prototype had a 14-mm-long needle with a 1.1 mm diameter, whereas the later design consisted of a 8Fr needle ablation catheter with an integrated retractable 27-G needle able to be extended up to 10 mm. This upgraded needle ablation catheter also provides the option of recording intramural uni- and bipolar signals, options for pacing from the needle to confirm functionally critical areas and a sensor for visualization in electro-anatomical mapping systems. The irrigation port allowed injection of contrast dye prior to ablating to confirm appropriate positioning and tissue staining. In animal studies, contrast application in the apical segments has been shown to not rarely cause pericardial staining though without adverse events [75].

First in-human feasibility studies in 2013 including 8 refractory VT patients demonstrated intramyocardial VT mapping, energy delivery was possible and a mean of 22 needle ablation lesions per patient were delivered. Yet one cardiac tamponade and 2 complete heart blocks after ablation in the basal septum occurred, and at 12 months, 50% of the patients had recurrent VT [76]. To improve the selection of optimal target sites, recordings of intramural bipolar EGM and pace mapping over the needle were employed in a subsequent case series [77]. In a FDA investigational device exemption trial that included 31 patients with 70% non-ischemic substrates, a median of 15 needle lesions/patient were delivered with one pericardial effusion requiring a percutaneous drain. At 6 months follow-up, 48% were free of recurrent arrhythmias, and another 19% improved [25]. A subsequent publication involving 58 needle ablation procedures in complex VT patients (48% combined with half normal saline, 74% combined with conventional endocardial RFA) reported acute success for PVC abolition of 74% and non-inducible reentry VT in 49% [78]. Most recently, the cumulative experience from treating 111 patients with refractory ventricular arrhythmias has shown an improved arrhythmia control in 78% of PVC, reduction in hospitalization in 69% of VT patients and abolition of VT in 47% [79••]. Despite the promising technology and acceptable safety profile, irrigated RF needle catheters are currently not available for routine clinical use.

A technique applying a similar concept is the percutaneous intramyocardial septal radiofrequency ablation (PIMSRA), which has been proposed for hypertrophic obstructive cardiomyopathy for outflow tract reduction. Access is gained by ultrasound guided over a left apical puncture with the RF catheter being placed intramyocardially in the septum and energy delivered to the anterior and/or inferior septum [71]. Whether this may represent a practical technique for VT ablation has not yet been evaluated and in non-hypertrophied myocardium may represent a greater challenge.

#### Saline Enhanced Radiofrequency (“SERF”) Needle

A variation of the above-described RF needle uses heated saline, a technique pioneered in 2014 and involving irrigation with 80° saline at 20 ml/min over 24 radial side holes of a prototype ablation needle catheter. The synergistic effect to create large thermal lesions has been tested in an infarct animal model with lesions of on average 18 mm depth and 11 mm width [80]. A follow-up study using a 8Fr unidirectional quadripolar ablation catheter with a 4-mm tip and 25-G retractable needle reconfirmed the ability to create larger, homogenous and more transmural lesions. The benefits were more evident if employed in thick-walled myocardium with less loss of injected heated saline. A total of 5 ventricular fibrillation episodes were observed during 15 SERF applications in the dog heart, but no steam pops, perforation or epicardial haemorrhage [81]. As of today, no in-human use of SERF needles has been published.

#### Multipolar Linear RF Catheter

A linear catheter design would allow rapid delivery of multiple continuous lesions in a linear pattern on the endo- and epicardial ventricular surface to generate lines of block across functionally critical areas. A preclinical study investigated the use of the straightened-out multipolar RF ablation catheter (originally designed for PVI with a circular design) containing 7 irrigated electrodes spaced over 3.5 cm and delivering up to 25 W simultaneously to each electrode. In an animal study, epicardial linear lesions were longer and with a larger volume than focal point-by-point ablation but no differences in the endocardial lines. There were no gaps within the ablation lines of the linear catheter but up to 53% of focal lines highlighting the ability to deliver contiguous lesions in a single ablation [70]. These findings have not yet been translated into the use of a linear ablation catheter in humans.

#### Spherical RF Ablation Catheter

The Sphere-9™ catheter (Affera, Inc.) has a 9-mm expandable spherical monopolar irrigated RF tip with 9 mini surface electrodes to record EGMs but also measure surface temperature. The contact surface may be up to tenfold greater relative to standard catheters. In animal studies, the spherical RF catheter created significantly larger lesions in all dimensions of up to 10.3 ± 2.9 mm depth and 15.8 mm ± 3 mm length when compared to the standard 3.5-mm tip RF catheter with no safety events [82, 83]. More recently the Sphere-9 catheter has been promoted as a combined RF and pulse field (PF) catheter for atrial fibrillation [84] and is now integrated into the Affera mapping and navigation platform (acquired by Medtronic in 2022). The multi-centre randomized SPHERE Per-AF trial (NCT05120193) is ongoing, yet currently, no investigations for its use in the VT are reported.

#### Partially Insulated RF Ablation Catheter Tips (PIFA)

In order to direct RF current preferentially to the targeted cardiac tissue, the use of a thermally conductive electrical insulation, causing a very high electrical impedance, to partially coat the catheter tip has been proposed. Preclinical studies demonstrated higher temperatures and lesion depth with larger impedance changes but also higher steam pop rates. The latter was mitigated by external irrigation, a larger electrode and more thermally conductive insulation [67]. Further improvements in design include added fenestration to the coating for passive cooling allowing for larger ablation lesion volumes without temperature limitations [85]. Other designs for partially insulated catheters for percutaneous epicardial ablation have been proposed and combined with intra-pericardial ultrasound via interspersed windows to enable visualization of the epicardial structures, controlling the directionality of energy and protecting adjacent structures all at the same time [86]. Despite the biophysiological compelling rational of this catheter design and promising preclinical data, this concept has not yet been translated and applied in any in vivo studies.

#### Intramyocardial RF Wire and Coil Embolization

Case reports have described the feasibility of applying radiofrequency over either trans-coronary or trans-venously inserted and intentionally perforating guidewire to the intramyocardial target zone [68, 87]. The proximal end of the wire is placed in a water bath together with the ablation catheter transmitting the energy to the wire. Alternatively, intracoronary wire mapping has been used to guide and deliver coil embolization [88]. The techniques are derived from interventional cardiology procedures like CTO revascularizations and employ commercially available coronary angioplasty equipment. Only isolated reports have been published for their use for VT ablation.

### Practical Considerations

Despite a number of innovative approaches and encouraging (pre)clinical findings regarding lesion depths and width and within that context reasonable safety profiles, none of the above-mentioned RF devices is clinically approved outside of trials. Further evidence, experience and more widespread accessibility and availability of these devices are required to establish long-term efficacy and safety profile in a larger cohort of VT patients to better define their role as an alternative and/or complementing approach to available RF techniques.

## Conclusion

In elective procedures, careful preprocedural planning may help to identify patients in whom standard RF ablation may be insufficient to reach the presumed critical areas, and advanced RF strategies should be considered. Recent clinical experience with these investigational approaches underlines the fine balance between wider and deeper lesions and increased safety concerns. Patient counselling and consent, local expertise and available equipment for advanced RF strategies should determine the choice of the ablation strategy respectively technology. Further refinement of RF catheter designs and individualized ablation settings based on substrate and patient characteristics for each technology may improve efficacy and safety in the future.

## Abbreviations

VT: Ventricular tachycardia
ICD: Implantable cardioverter/defibrillator
LV: Left ventricle
RV: Right ventricle
LA: Left atrium
LVEF: Left ventricular ejection fraction
CS: Coronary sinus
ICM: Ischemic cardiomyopathy
NICM: Non-ischemic cardiomyopathy
ACM: Arrhythmogenic cardiomyopathy
HCM: Hypertrophic cardiomyopathy
TOE: Transoesophageal echocardiography
CT: Computer tomography
EGM: Electrogram
LAO: Left anterior oblique
RAO: Right anterior oblique
LAT: Local activation time
CO_2_: Carbon dioxide
ACT: Activated clotting time
CL: Cycle length
